# Decrease in serum levels of autotaxin in COVID-19 patients

**DOI:** 10.1080/07853890.2022.2143554

**Published:** 2022-11-11

**Authors:** Takuya Shimura, Makoto Kurano, Koh Okamoto, Daisuke Jubishi, Hideki Hashimoto, Kuniyuki Kano, Koji Igarashi, Satoshi Shimamoto, Junken Aoki, Kyoji Moriya, Yutaka Yatomi

**Affiliations:** a Department of Clinical Laboratory, The University of Tokyo Hospital, Tokyo, Japan; b Department of Clinical Laboratory Medicine, Graduate School of Medicine, The University of Tokyo, Tokyo, Japan; c Department of Infectious Diseases, The University of Tokyo Hospital, Tokyo, Japan; d Department of Health Chemistry, Graduate School of Pharmaceutical Sciences, The University of Tokyo, Tokyo, Japan; e Bioscience Division, TOSOH Corporation, Kanagawa, Japan

**Keywords:** Autotaxin, lysophosphatidic acids, COVID-19, severity, inflammation

## Abstract

**Introduction:**

In order to identify therapeutic targets in Coronavirus disease 2019 (COVID-19), it is important to identify molecules involved in the biological responses that are modulated in COVID-19. Lysophosphatidic acids (LPAs) are involved in the pulmonary inflammation and fibrosis are one of the candidate molecules. The aim of this study was to evaluate the association between the serum levels of autotaxin (ATX), which are enzymes involved in the synthesis of lysophosphatidic acids.

**Material and methods:**

We enrolled 134 subjects with COVID-19 and 58 normal healthy subjects for the study. We measured serum ATX levels longitudinally in COVID-19 patients and investigated the time course and the association with severity and clinical parameters.

**Results:**

The serum ATX levels were reduced in all patients with COVID-19, irrespective of the disease severity, and were negatively associated with the serum CRP, D-dimer, and anti-severe acute respiratory syndrome coronavirus 2 (SARS-CoV-2) antibody levels.

**Discussion:**

Considering the biological properties of LPAs in the pulmonary inflammation and fibrosis, modulation of ATX might be compensatory biological responses to suppress immunological overreaction especially in the lung, which is an important underlying mechanism for the mortality of the disease.

**Conclusions:**

COVID-19 patients showed a decrease in the serum levels of ATX, irrespective of the disease severity.

**Key Messages**
- Autotaxin (ATX) is an enzyme involved in the synthesis of lysophosphatidic acid (LPA), which has been reported to be involved in pulmonary inflammation and fibrosis. Patients with COVID-19 show decrease in the serum levels of ATX. Modulation of ATX might be compensatory biological responses to suppress immunological overreaction.

## Introduction

Coronavirus disease 2019 (COVID-19), caused by severe acute respiratory syndrome coronavirus 2 (SARS-CoV-2), caused a worldwide pandemic in early 2020 with morbidity and mortality throughout the world. It is an important task to develop novel drugs to overcome this pandemic, as well as to protect the population by vaccination to prevent the spread of COVID-19. Although antiviral agents have been expected, one of the important goals of treatment is to control the biological response to the infection. One of the characteristics of COVID-19 is that the disease has diverse phenotypes, and the inadequate immunological overreaction, namely, cytokine storm, and organ injuries arising thereof have been deemed to play critical pathophysiological roles in patients with severe COVID-19 [1,2]. In regard to drugs that can modulate immune responses, monoclonal antibodies against SARS-CoV-2 [3] and cytokines [4] have been shown to be promising, in addition to steroids [5].

A series of elegant studies has demonstrated that bioactive lipids are generated during inflammatory and immune responses and regulate them [6–9]. In addition, accumulating data suggest that lysophospholipid mediators, such as LPA, are also generated during inflammatory and immune responses and regulate the progression of disease states both positively and negatively, depending on each specific G protein-coupled receptor [10]. Although research is limited at present, several studies have used lipidomics approaches to investigate the significant associations [11–18]. In this study, as proteins related with lysophospholipids, we investigated the serum levels of autotaxin (ATX) in patients with COVID-19.

ATX is an enzyme involved in the production of LPA [19], a group of potent lysophospholipid mediators, that exerts its bioactivities through six kinds of G protein-coupled receptors (LPA_1_–_6_) located on the cell membrane [20]. The pathological roles of LPA and ATX in human diseases have been well studied. LPA and ATX have been reported to be involved in inflammatory diseases, such as rheumatoid arthritis [21,22], Graves’ disease [23], and cancer-related inflammation [24]. Moreover, ATX and LPA have been reported to play crucial roles in pulmonary inflammation and fibrosis [25,26], which are the main causes of the acute respiratory failure and other complications in COVID-19.

In this report, we present a detailed analysis of the changes of the serum ATX levels in patients with COVID-19.

## Materials and methods

### Subjects

We collected residual serum samples remaining after routine clinical testing from 134 subjects who had been diagnosed as having COVID-19 based on the results of RT-PCR between April 2020 and January 2021. None of the subjects had been vaccinated against SARS-CoV-2. Seven subjects were asymptomatic and 127 subjects were symptomatic. The symptomatic subjects were classified into three groups according to the disease severity: severity level 1 (mild disease group; did not require oxygen therapy), severity level 2 (moderate disease group; required oxygen therapy, but not mechanical ventilatory support) and severity level 3 (severe disease group; required mechanical ventilatory support). Subjects with complications such as cirrhosis, hyperthyroidism and pregnancy were excluded, since the presence of these complications could potentially affect the serum levels of ATX [23,27,28]. The characteristics of the subjects are described in Table 1. As a control group, serum samples were collected from 58 healthy adult volunteers after obtaining their written informed consent for participation in the study. To obtain the serum samples, whole blood samples were directly collected into glass tubes and allowed to stand for 15 min at room temperature to allow blood clotting; the serum was then separated by centrifugation at 1500 ×*g* for 5 min.

**Table 1.** Characteristics of the subjects enrolled in this study.

	Asymptomatic	Severity level 1	Severity level 2	Severity level 3	Healthy subjects
Age (mean ± SD)*	56.6 ± 25.2	54.0 ± 18.7	65.0 ± 12.8	67.5 ± 11.9	31.1 ± 10.4
Sex (male/female, *n*)	1/6	23/18	45/17	19/5	25/33
Diabetes mellitus (%)	14.3	22.0	37.1	37.5	–
Hypertension (%)*	28.6	26.8	54.8	54.2	–
Current smoking (%)	0	14.6	17.7	16.7	–
Drugs used:					
Favipiravir (%)*	14.3	39.0	45.2	54.2	–
Nafamostat (%)*	0	12.2	22.6	54.2	–
Remdesivir (%)*	0	4.9	51.6	33.3	–
Steroids (%)*	0	12.2	62.9	87.5	–

Age is represented as the median ± SD. The differences in age among the three severity classes of COVID-19 were assessed using an independent Kruskal–Wallis test, followed by the Games Howell test for post-hoc analysis. The differences in the sex distribution, frequency of complications and rate of use of specific drugs among the three patient groups classified according to the severity of COVID-19 were assessed by the Chi-square test. *p < 0.01.

This study was performed in accordance with the ethical guidelines laid down in the Declaration of Helsinki. Written informed consent for sample analysis was obtained from some patients; other participants from whom written informed consent could not be obtained as they had been discharged or transferred from the hospital, were informed about the study and informed consent was obtained in the form of an opt-out on the website. Patients who were unwilling to be enrolled in our study were excluded. The study design was approved by The University of Tokyo Medical Research Center Ethics Committee (2602 and 2020206NI).

The day after onset of COVID-19 was determined by the date of the symptom onset reported by the patients. Since the timing when RT-PCR tests were performed largely varied among patients, we used the day after the symptom onset to investigate the modulation of serum ATX levels in the time course of COVID-19.

### Measurement of ATX levels

The ATX antigen levels were determined using a two-site immunoenzymometric assay with the TOSOH AIA system (TOSOH, Tokyo, Japan) [29]. The ATX levels were measured in the samples available at every 2 d. The number or the samples obtained for the measurements on specified days after the onset of COVID-19 are listed in Supplemental Table S1. This measurement system is validated in the previous article by us [29] and approved as an *in vitro* diagnostic for liver fibrosis in Japan [27].

### Measurements of serum anti-SARS-CoV-2 IgM and IgG, CRP and D-dimer levels

Anti-SARS-CoV-2 antibody testing was performed using the iFlash3000 fully automated chemiluminescent immunoassay analyzer (Shenzhen YHLO Biotech Co., Ltd., Shenzhen, China), as reported previously [30,31]. Serum CRP and D-dimer levels were measured by the latex agglutination immunoassay (LZ test ‘Eiken’ CRP-HG, Eiken Kagaku Company Limited, Tokyo, Japan; LPIA-ACE, Mitsubishi Chemical Medience Co, Tokyo, Japan).

### Statistical analysis

**Figure 1.**
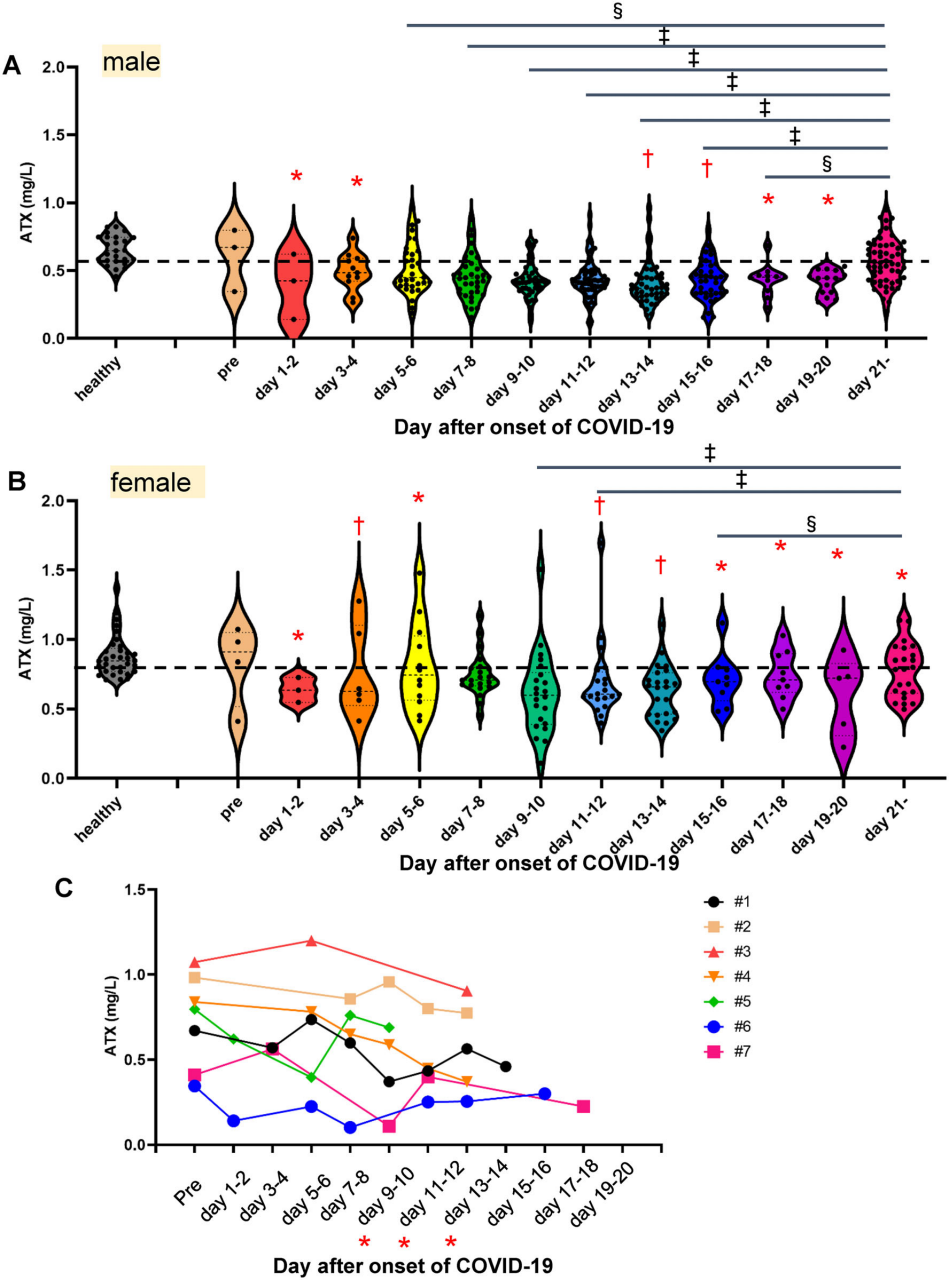
Serum ATX levels in COVID-19 patients. The serum ATX levels were measured in patients with COVID-19 (symptomatic, *n* = 127 [male, *n* = 87; female, *n* = 40]; asymptomatic, *n* = 6 [male, *n* = 1; female, *n* = 5]) and healthy subjects (*n* = 58 [male, *n* = 25; female, *n* = 33]). (A,B) Time-course of serum ATX levels in symptomatic COVID-19 patients and distribution of serum ATX levels in the healthy subjects (A, male; B, female) Differences in the serum ATX levels between healthy subjects and COVID-19 patients were assessed by the Mann–Whitney U test, *p < 0.01, ^†^*p* < 0.05 *vs.* healthy subjects. Differences between serum ATX levels measured on specified days after the onset of COVID-19 and those measured after day 21 in individual subjects were assessed by the Wilcoxon signed-rank sum test. ^‡^*p* < 0.01, ^§^*p* < 0.05 *vs.* level measured after day 21. The asymptomatic patients were not included in Figure 1(A,B). (C) Time-course of serum ATX levels in the COVID-19 patients for whom samples collected before the onset of the disease were available (*n* = 7). Differences between serum ATX levels measured on specified days after the onset of COVID-19 and those measured before symptom onset (Pre) in individual subjects were assessed by the Wilcoxon signed-rank sum test. *p < 0.05 *vs.* Pre.

**Figure 2.**
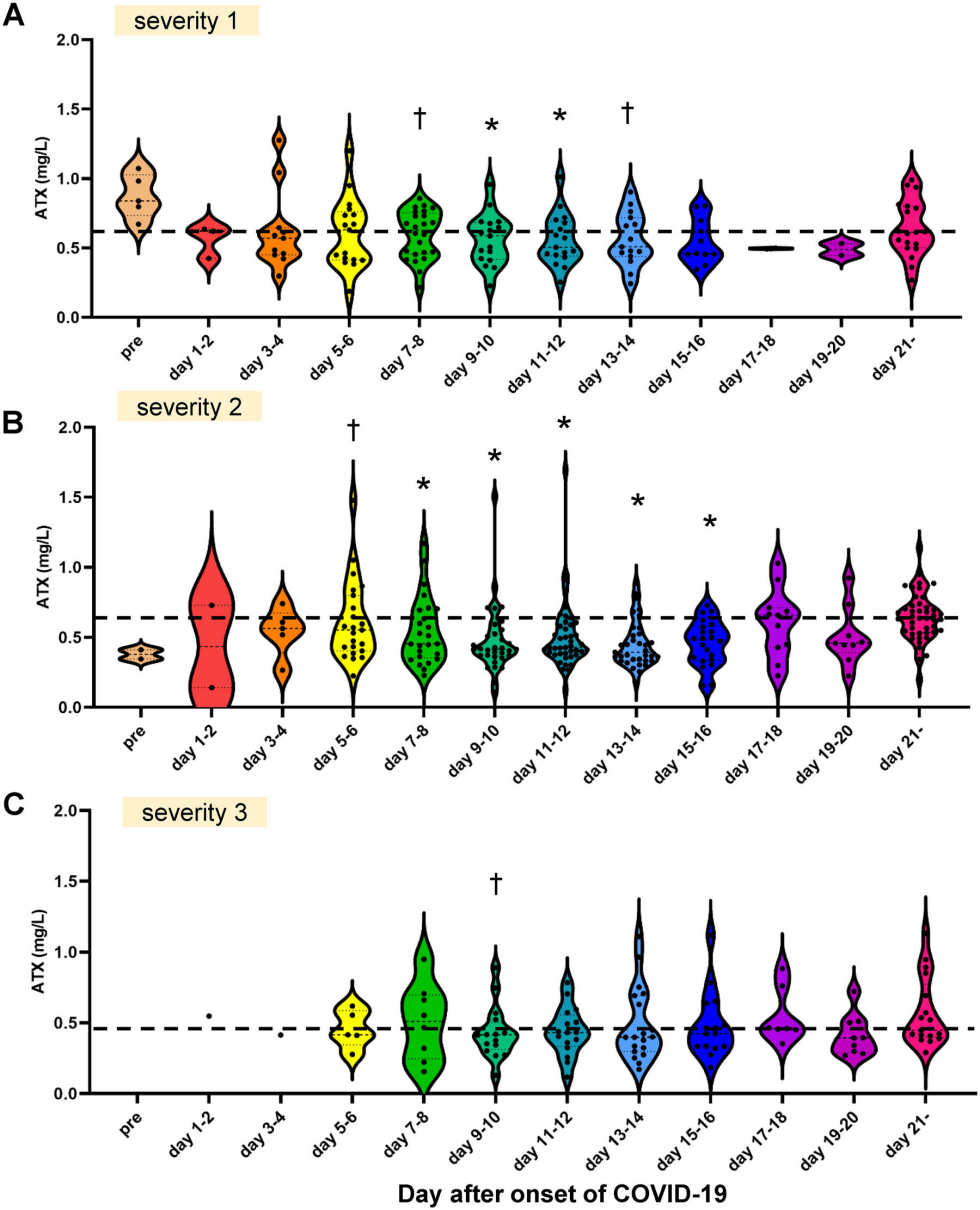
Associations of the time-course of the serum ATX levels with the severity of COVID-19. Associations between the serum ATX levels and severity of COVID-19 are presented. Time-course of serum ATX levels in the patients with mild COVID-19 (severity level 1) (A; *n* = 41), moderate COVID-19 (severity level 2) (B; *n* = 62) and severe COVID-19 (severity level 3) (C; *n* = 24). The asymptomatic patients are not included in Figure 2. Differences between the serum ATX levels measured on specified days after the onset of COVID-19 from those measured after day 21 in individual subjects were assessed by the Wilcoxon signed-rank sum test. *p < 0.01, ^†^*p* < 0.05 *vs.* level measured after day 21. The horizontal bar represents the means of serum ATX levels measured after day 21.

All the data were statistically analysed using SPSS (Chicago, IL). The results are expressed as dot plots. Differences between two groups were evaluated by the Mann − Whitney U test, differences among three independent groups were assessed by an independent Kruskal − Wallis test, followed by the Games Howell test for post-hoc analysis, correlations between two parameters were evaluated by Spearman’s correlation test, and difference between two paired groups were assessed by the Wilcoxon signed-rank sum test, since normality or equality of variance was rejected by the Kolmogorov − Smirnov test or the Levene test for most of the parameters. The differences in the frequency of complications and rate of use of specific drugs among the three severity classes of COVID-19 were assessed using a Chi-square test. The independent effects of clinical parameters and the results of clinical laboratory tests on the differences between specific points and after day 21 in individual subjects (⊿ATX = [ATX on specific day] – [ATX after day 21]) were investigated with multiple regression analyses using ⊿ATX as an objective variable and sex, age, severity of the disease, CRP, D-dimer, anti-SARS-CoV-2 IgM, anti-SARS-CoV-2 IgG, complete blood counts, AST, ALT, albumin, creatinine and coagulation tests as possible explanatory factors. *p* < 0.05 was regarded as denoting statistical significance in all the analyses. The numbers of samples obtained on specified days after the onset to compare the values with those measured after day 21 in individual subjects (Figures 1(A,B) and 2) are listed in Supplemental Table S2.

**Table 2.** Correlations of the serum ATX levels with clinical parameters in male patients with COVID-19.

Day	Anti-SARS-CoV-2 IgM (A.U.)	Anti-SARS-CoV-2 IgG (A.U.)	CRP (mg/L)	D-dimer (mg/L)
Day 1–2	*r* = −0.500, *p* = 0.667, *n* = 3	*r* = −0.500, *p* = 0.667, *n* = 3	*r* = −0.500, *p* = 0.667, *n* = 3	n.d., *n* = 1
Day 3–4	*r* = 0.661, *p* = 0.027, *n* = 11	*r* = 0.164, *p* = 0.631, *n* = 11	*r* = −0.347, *p* = 0.327, *n* = 10	*r* = 0.027, *p* = 0.949, *n* = 9
Day 5–6	*r* = −0.042, *p* = 0.817, *n* = 33	*r* = −0.028, *p* = 0.879, *n* = 33	*r* = −0.079, *p* = 0.680, *n* = 30	*r* = 0.297, *p* = 0.135, *n* = 27
Day 7–8	*r* = −0.457, *p* = 0.005, *n* = 36	*r* = −0.294, *p* = 0.082, *n* = 36	*r* = −0.097, *p* = 0.584, *n* = 34	*r* = −0.186, *p* = 0.292, *n* = 34
Day 9–10	*r* = −0.313, *p* = 0.039, *n* = 44	*r* = −0.502, *p* = 0.001, *n* = 44	*r* = −0.198, *p* = 0.204, *n* = 44	*r* = −0.405, *p* = 0.009, *n* = 41
Day 11–12	*r* = −0.173, *p* = 0.201, *n* = 56	*r* = −0.260, *p* = 0.053, *n* = 56	*r* = −0.163, *p* = 0.234, *n* = 55	*r* = −0.247, *p* = 0.078, *n* = 52
Day 13–14	*r* = −0.130, *p* = 0.389, *n* = 46	*r* = −0.137, *p* = 0.362, *n* = 46	*r* = −0.054, *p* = 0.724, *n* = 45	*r* = −0.243, *p* = 0.112, *n* = 44
Day 15–16	*r* = 0.151, *p* = 0.359, *n* = 39	*r* = −0.165, *p* = 0.315, *n* = 39	*r* = −0.180, *p* = 0.280, *n* = 38	*r* = −0.354, *p* = 0.032, *n* = 37
Day 17–18	*r* = −0.427, *p* = 0.190, *n* = 11	*r* = −0.345, *p* = 0.298, *n* = 11	*r* = −0.009, *p* = 0.979, *n* = 11	*r* = −0.027, *p* = 0.937, *n* = 11
Day 19–20	*r* = −0.279, *p* = 0.314, *n* = 15	*r* = 0.075, *p* = 0.790 *n* = 15	*r* = −0.451, *p* = 0.092, *n* = 15	*r* = −0.483, *p* = 0.080, *n* = 14

Correlations between two parameters were evaluated by Spearman’s correlation test.

## Results

### Serum ATX levels were reduced in patients with COVID-19

First, we compared the serum ATX levels between healthy subjects and COVID-19 patients on each day after the onset of COVID-19. Since the serum ATX levels were higher in female than in male patients [29], we analysed the results in the male and female patients separately. In the male patients, the serum ATX levels measured on day 1 − 2 and day 3 − 4, and from day 13 − 14 to day19 − 20 were significantly lower as compared to those measured on the same days in healthy subjects (Figure 1(A)); in the female patients, the serum ATX levels measured on day 1 − 2, day 3 − 4, day 5 − 6, day 11 − 12, day 15 − 16, day 17 − 18 and day 19 − 20 were significantly lower than those measured on the same days in healthy subjects (Figure 1(B)). In the subjects for whom samples collected prior to the onset of COVID-19 were available, the serum ATX levels measured on day 9 − 10, day 11 − 12 and day 13 − 14 were significantly lower as compared to the values measured prior to the onset of COVID-19 (Figure 1(C)). We also compared the serum ATX levels measured on specified days after the onset of symptoms and those measured later than 21 d after onset of the disease in individual subjects. The serum ATX levels measured from day 5 − 6 to day 17 − 18 in the male patients and from day 9 − 10 to day15 − 16 in the female patients were significantly lower than those measured later than 21 d after the disease onset. In regard to asymptomatic subjects, the time-course is shown in Supplemental Figure S1. Serum ATX levels measured from day1 − 2 to day 9 − 10 were not significantly different between asymptomatic female COVID-19 patients and healthy female subjects.

Steroid treatment, sometimes used to treat COVID-19 patients, can decrease the serum ATX levels [32]. To avoid possible confounding of the analysis results, we analysed the results separately in patients treated and not treated with steroids. As shown in Supplemental Figures S2 and S3, we obtained similar results between the subjects treated and not treated with steroids. These results suggest that steroid treatment has no effect on the alterations of the serum ATX levels in patients with COVID-19.

**Figure 3.**
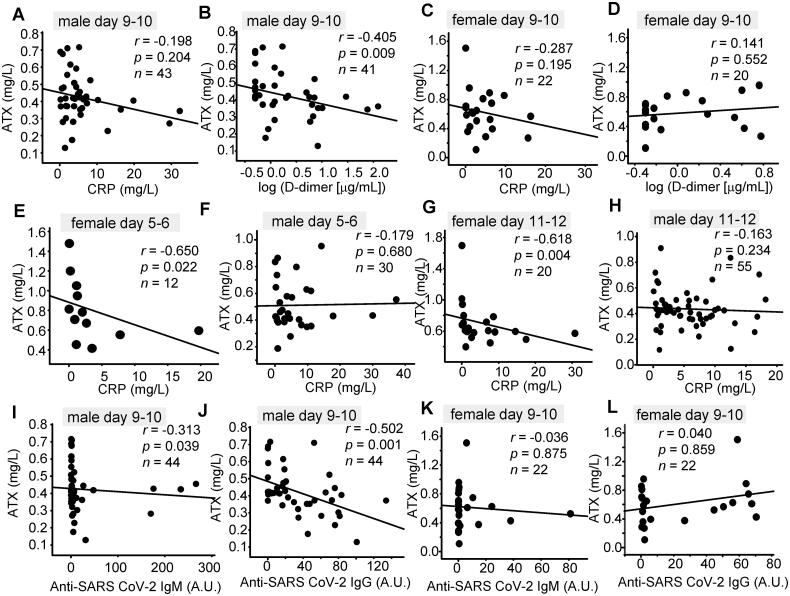
Correlations of the serum ATX levels with clinical parameters. Correlations between the serum ATX levels and the serum CRP, D-dimer, and anti-SARS-CoV-2 antibody levels are shown. (A, C, E–H) Correlations between serum ATX levels and serum CRP levels measured on day 5–6 (E, F), day 9–10 (A, C) and day 11–12 (G, H) in male (A, E, G) and female (C, F, H) patients. (B, D) Correlations between serum ATX levels and serum D-dimer levels measured on day 9–10 in male (B) and female (D) patients. (I–L) Correlations between serum ATX levels and serum anti-SARS-CoV-2 IgM (I, K)/anti-SARS-CoV-2 IgG (J, L) levels measured on day 9–10 in male (I, J) and female (K, L) patients. Correlations between two parameters were evaluated by Spearman’s correlation test.

### Serum ATX levels were not apparently associated with the severity of COVID-19

In regard to the association of the serum ATX levels with the severity of COVID-19, the serum ATX levels were significantly lower on several specified days after symptom onset as compared to those measured after day 21 in all the three groups classified according to the disease severity (Figure 2). Although the difference between the serum ATX levels in the specific day and day after day 21 seemed small in the subjects with severity 3, we observed no significant differences in the serum ATX levels on any specified days among the three groups classified according to the disease severity. When we calculated the differences between specific points and after day 21 in individual subjects (⊿ATX = [ATX on specific day] – [ATX after day21]), we observed no statistically significant differences among three severity groups (Supplemental Figure S4).

**Figure 4.**
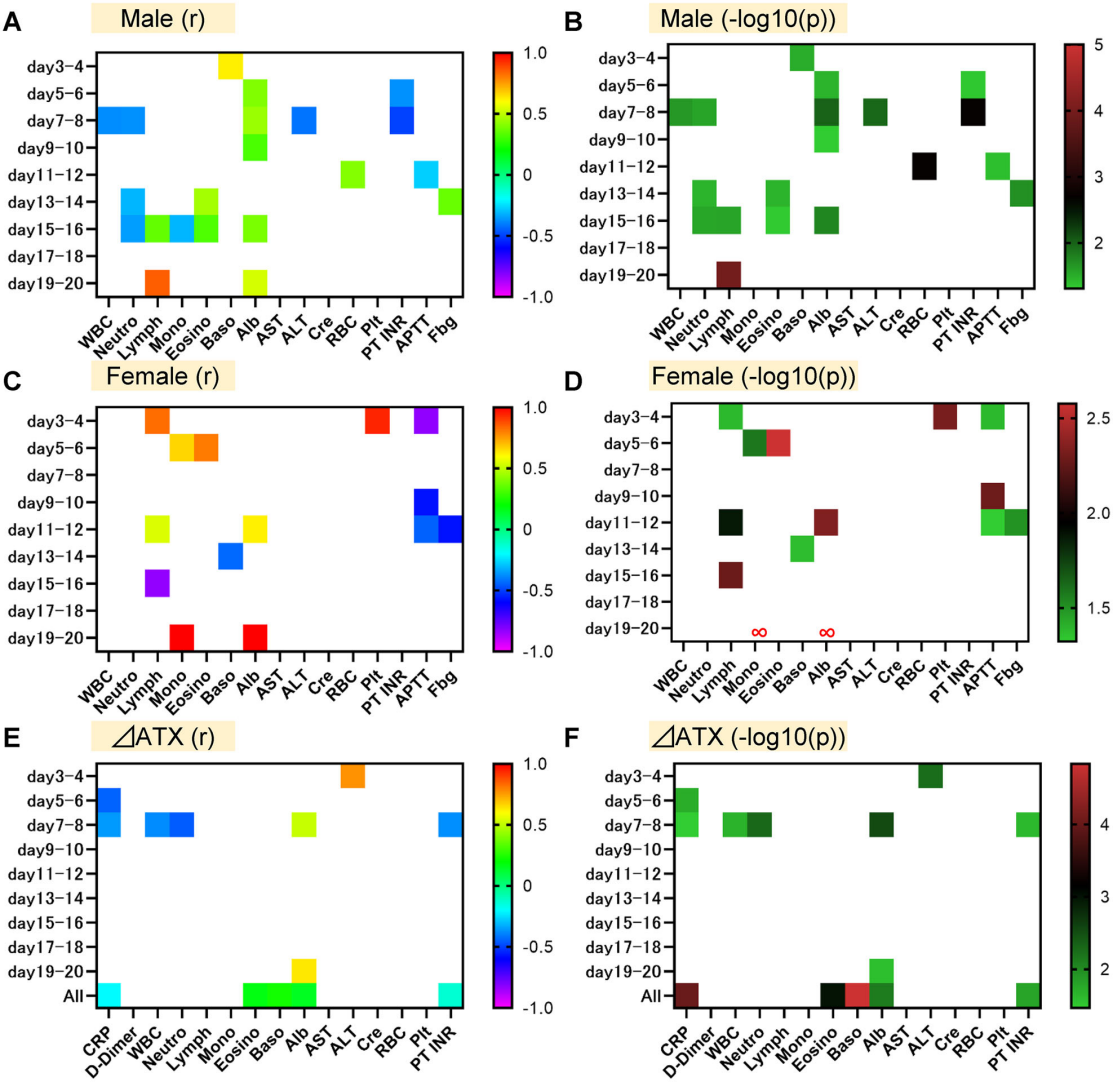
Correlations of serum ATX levels with the results of clinical laboratory tests. Spearman rank correlation analyses were used to compare serum ATX levels (A–D) or ⊿ATX ([ATX on specific day] – [ATX after day 21]) (E, F) with the results of various clinical laboratory tests in samples collected at the specific time points. Correlation coefficients (A, C, E) and *p* values (shown as − log10 [*p* value]) (B, D, F) are shown as a heat map. The correlation coefficients and *p* values of non-significant results are shown as blanks. (A, B) Male subjects. Day 3–4, *n* = 12; day 5–6, *n* = 31; day 7–8, *n* = 36; day 9–10, *n* = 45; day 11–12, *n* = 56; day 13–14, *n* = 46; day 15–16, *n* = 39; day 17–18, *n* = 11; day 19–20, *n* = 15. (C, D) Female subjects. Day 3–4, *n* = 6; day 5–6, *n* = 12; day 7–8, *n* = 21; day 9–10, *n* = 22; day 11–12, *n* = 20; day 13–14, *n* = 23; day 15–16, *n* = 9; day 17–18, *n* = 9; day 19–20, *n* = 4. (E, F) Both male and female subjects. Day 3–4, *n* = 11; day 5–6, *n* = 28; day 7–8, *n* = 36; day 9–10, *n* = 45; day 11–12, *n* = 51; day 13–14, *n* = 45; day 15–16, *n* = 35; day 17–18, *n* = 19; day 19–20, *n* = 13.

### Serum ATX levels were negatively associated with the serum CRP, D-dimer and anti-SARS-CoV-2 antibody levels on several prespecified days after the onset of COVID-19

Next, we compared the associations of the serum ATX levels with the clinical parameters. As shown in Tables 2 and 3 and Figure 3, the serum ATX levels showed significant negative correlations with the serum CRP levels on day 5 − 6 and day 11 − 12 in female patients, and with the serum D-dimer levels on day 9 − 10 and day 15 − 16 in male patients, and on day 3 − 4 in the female patients. In regard to the association with the anti-SARS-CoV-2 antibody levels, serum ATX levels showed significant negative correlations with the anti-SARS-CoV-2 IgM antibody levels measured on day 7 − 8 and day 9 − 10 in the male patients and on day 13 − 14 in the female patients, although significant positive correlations were observed with the serum anti-SARS-CoV-2 IgM levels measured on day 3 − 4 in a limited number of male patients. The serum ATX levels also showed significant negative correlations with the anti-SARS-CoV-2 IgG levels measured on day 9 − 10 in the male patients.

**Table 3.** Correlations of the serum ATX levels with clinical parameters in female patients with COVID-19.

Day	Anti-SARS-CoV-2 IgM (A.U.)	Anti-SARS-CoV-2 IgG (A.U.)	CRP (mg/L)	D-dimer (mg/L)
Day 1–2	*r* = −0.500, *p* = 0.667, *n* = 3	*r* = −0.500, *p* = 0.667, *n* = 3	*r* = −0.500, *p* = 0.667, *n* = 3	*r* = −1.000, *p* n.d., *n* = 2
Day 3–4	*r* = 0.029, *p* = 0.957, *n* = 6	*r* = −0.257, *p* = 0.623, *n* = 6	*r* = −0.771, *p* = 0.072, *n* = 6	*r* = −0.845, *p* = 0.034, *n* = 6
Day 5–6	*r* = −0.018, *p* = 0.957, *n* = 12	*r* = −0.126, *p* = 0.697, *n* = 12	*r* = −0.650, *p* = 0.022, *n* = 12	*r* = −0.410, *p* = 0.240, *n* = 10
Day 7–8	*r* = −0.138, *p* = 0.552, *n* = 21	*r* = −0.181, *p* = 0.434, *n* = 21	*r* = −0.110, *p* = 0.636, *n* = 21	*r* = −0.006, *p* = 0.978, *n* = 21
Day 9–10	*r* = −0.036, *p* = 0.875, *n* = 22	*r* = 0.040, *p* = 0.859, *n* = 22	*r* = −0.287, *p* = 0.195, *n* = 22	*r* = 0.141, *p* = 0.552, *n* = 20
Day 11–12	*r* = −0.229, *p* = 0.332, *n* = 20	*r* = −0.227, *p* = 0.336, *n* = 20	*r* = −0.618, *p* = 0.004, *n* = 20	*r* = −0.193, *p* = 0.427 *n* = 19
Day 13–14	*r* = −0.504, *p* = 0.014, *n* = 23	*r* = −0.067, *p* = 0.761, *n* = 23	*r* = −0.027, *p* = 0.907, *n* = 21	*r* = 0.113, *p* = 0.635, *n* = 20
Day 15–16	*r* = −0.483, *p* = 0.187, *n* = 9	*r* = 0.250, *p* = 0.516, *n* = 9	*r* = 0.238, *p* = 0.570, *n* = 8	*r* = 0.491, *p* = 0.217, *n* = 8
Day 17–18	*r* = 0.357, *p* = 0.385, *n* = 8	*r* = 0.095, *p* = 0.823, *n* = 8	*r* = −0.233, *p* = 0.546, *n* = 9	*r* = −0.017, *p* = 0.966, *n* = 9
Day 19–20	*r* = −0.100, *p* = 0.873, *n* = 5	*r* = −0.100, *p* = 0.873, *n* = 5	*r* = −0.300, *p* = 0.624, *n* = 5	*r* = −0.700, *p* = 0.188, *n* = 5

Correlations between two parameters were evaluated by Spearman’s correlation test.

### Correlations between serum ATX levels and the results of routine laboratory tests

Lastly, to investigate the mechanisms for the decrease in serum ATX levels in COVID-19, we investigated the correlations between serum ATX levels and the results of routine laboratory tests. Figure 4(A,B) shows the results in male subjects and Figure 4(C,D) shows those in female subjects. Among complete blood counts, generally, serum ATX levels showed significant negative correlations with neutrophil counts and positive ones with lymphocyte and eosinophil counts. At some points, serum ATX levels had positive correlations with RBC and platelet counts. Regarding monocyte and basophil counts, the associations were not consistent. Among clinical chemistry tests, serum ATX levels had positive correlations with albumin levels, while they had negative ones with ALT in day 7 − 8 in male subjects. Among coagulation tests, serum ATX levels had negative correlations with PT-INR and APTT, whereas the correlations with fibrinogen were not consistent.

Next, we investigated the correlations between ⊿ATX ([ATX on specific day] – [ATX after day 21]) and the results of routine laboratory tests (Figure 4(E,F)). In day 3 − 4, a positive correlation was observed between ⊿ATX and ALT. ⊿ATX had negative correlations with CRP in day 7 − 8 and with WBC, neutrophil counts, and PT-INR in day 5 − 6. ⊿ATX had positive correlations with albumin in day 5 − 6 and day 19 − 20. When all the samples were analysed together, ⊿ATX showed negative correlations with CRP and PT-INR, while it showed positive ones with eosinophil and basophil counts and albumin.

We further performed multiple regression analyses for ⊿ATX. As shown in Table 4, platelet counts were selected as a negative explanatory factor in day 5 − 6, albumin, PT-INR, and AST as positive ones in day 7 − 8, and D-dimer as a positive one in day 13 − 14. When we analysed all the samples, creatinine and female are selected as positive explanatory variables.

**Table 4.** Multiple regression analyses for the difference between serum ATX levels at specific time points and those after day 21.

	Selected variables	Standardized *β*	*p* Value	*R* ^2^
Day 3–4	Not selected			
Day 5–6	Platelets	−0.539	0.021	0.290
Day 7–8	Alb	0.767	0.000	0.473
	PT INR	0.490	0.008	
	AST	0.411	0.028	
Day 9 − 10	Not selected			
Day 11 − 12	Not selected			
Day 13 − 14	D-Dimer	0.373	0.032	0.139
Day 15 − 16	Not selected			
Day 17 − 18	Not selected			
Day 19–20	Not selected			
All	Cre	0.213	0.001	0.059
	sex (female)	0.129	0.043	

The independent effects of clinical parameters and the results of clinical laboratory tests on the differences between specific points and after day 21 in individual subjects (⊿ATX = [ATX on specific day] – [ATX after day21]) were investigated with multiple regression analyses using ⊿ATX as an objective variable and sex, age, severity of the disease, CRP, D-dimer, anti-SARS-CoV-2 IgM, anti-SARS-CoV-2 IgG, complete blood counts, AST, ALT, albumin, creatinine and coagulation tests as possible explanatory factors. Day 3–4, *n* = 11; Day 5–6, *n* = 28; Day 7–8, *n* = 36; Day 9–10, *n* = 45; Day 11–12, *n* = 51; Day 13–14, *n* = 45; Day 15–16, *n* = 35; Day 17–18, *n* = 19; Day 19–20, *n* = 13.

## Discussion

In this study, we investigated the alterations in the levels of ATX, since it is an important task to identify the possible target molecules modulated in the body in response to SARS-CoV-2 infection, in which biological responses to the infection appear to play crucial roles in the pathogenesis, especially in determining the severity of the disease. We found decrease of the serum ATX levels in the patients with COVID-19, irrespective of the disease severity (Figures 1 and 2). Serum ATX levels are reported to be elevated in subjects with chronic liver diseases, especially HCV hepatitis [27], various types of cancers [33–35], pregnancy [28], Graves’ disease [23], rheumatoid arthritis [21,22] and lung diseases [25,6]; furthermore, steroids also known to reduce the serum ATX levels [32], besides a malnutrition state [36]. In COVID-19 patients, while use of steroids could affect the serum ATX levels [32], we performed stratified analyses, and found that steroids exerted no effect on the serum ATX levels (Supplemental Figures S2 and S3). Another possibility is malnutrition [36] due to the illness in COVID-19. As shown in Figure 2, however, decreased serum ATX levels were observed even in patients with mild COVID-19 and no obvious differences were observed among the three patient groups classified according to the severity of COVID-19 (Figure 2). Homeostasis of ATX has been well studied; the main source of circulating ATX is subcutaneous adipose tissue [37] and ATX is rapidly metabolized by the liver sinusoidal endothelial cells [38]. However, the persistent decrease of the serum ATX levels up to day 19 − 20 or later in the COVID-19 patients (Figure 1) suggested that some long-term mechanisms might be involved in the modulation of ATX in COVID-19. Actually, ACE2, a receptor for SARS-CoV-2, is well expressed in adipose tissue [39], which is the main source for ATX.

Although we could not elucidate the underlying mechanisms in this study, we investigated the association between the serum ATX levels and ⊿ATX and the results of laboratory tests with correlation analyses (Figure 4) and multiple regression analyses (Table 3). Regarding liver functions, ALT had a positive correlation with ⊿ATX in day 3 − 4 and AST was selected as a significant positive explanatory factor for ⊿ATX in day 7 − 8, which were consistent with the established modulations of serum ATX levels in the subjects with liver dysfunctions [27]. On the contrary, serum ATX had a negative correlation with ALT in day 7 − 8 in male subjects. Considering that serum ATX levels increased in the subjects with liver injuries [40], liver injuries which could accompany COVID-19 might not directly decrease serum ATX levels. Instead, CRP had negative correlations with serum ATX levels or ⊿ATX, suggesting that inflammation might negatively influence serum ATX levels in COVID-19.

Among complete blood counts, serum ATX levels showed significant negative correlations with neutrophil counts and positive ones with lymphocyte and eosinophil counts. ⊿ATX also had a negative correlation with neutrophil counts in day 7 − 8. Since neutrophil counts increase and lymphocyte and eosinophil counts decrease in the subjects treated with steroids, the downregulation of serum ATX levels by steroid [32] could explain these correlations. Platelet counts were selected as a negative exploratory factor for ATX in day 5 − 6. Although platelets were reportedly associated with ATX [41], the main source of ATX was not platelets, but adipose tissue [37]. Therefore, several confounding factors, such as inflammation, might exist. Serum ATX and ⊿ATX generally had positive correlations with albumin. Since inflammation could expend albumin and negative associations were observed between ATX or ⊿ATX and CRP, inflammation could explain the positive associations of ATX with albumin. Malnutrition, which could decrease both ATX(36) and albumin, might be another possible confounding factor. The selection of creatinine as a positive explanatory variable for ⊿ATX is difficult to explain at present, since ATX has not been demonstrated to be cleared through kidneys. Interestingly, the negative correlations were observed between ATX and PT-INR or APTT and D-dimer was selected as a positive exploratory factor for ⊿ATX in day 13 − 14. Although the association of ATX or LPA with DIC has not been established, several studies have demonstrated that ATX might promote pro-coagulative pathological conditions [41–43].

In regard to the pathophysiological significance of the decline of the serum ATX levels in COVID-19, ATX and LPA have been reported to be positively involved in the pathogenesis of inflammation, and this modulation might, in general, prevent the progression of COVID-19. Moreover, inhibition of ATX and the LPA axis protects against pulmonary inflammation and fibrosis [25], which cause respiratory failure in COVID-19 patients. A recent study also revealed that ATX is associated with ventilator-induced lung injury [26]. Although no significant differences in the serum ATX levels and ⊿ATX were observed among the three groups classified according to the COVID-19 severity, the negative correlations of the serum ATX levels with the serum CRP and D-dimer levels on several of the specified days after the onset of infection (Figure 3 and Tables 2 and 3) suggested the possibility that the decline of the serum ATX levels might represent a compensatory response to protect against COVID-19. However, in the fields other than pulmonary inflammation, anti-inflammatory properties of LPA have been proposed: LPA5 negatively modulates the immune responses by inhibiting the antigen-dependent activation of the B-cell and T-cell receptor [44–47]. LPA6 also reportedly suppresses the chemotaxis and tumour infiltration of CD8-positive T cells [48]. Concordant with the anti-inflammatory aspects of ATX, Serum ATX levels were also negatively correlated with the serum anti-SARS-CoV-2 IgM or IgG levels on several of the specified days after the onset of COVID-19, although several confounding factors might exist [49,50]. The further investigation on the association between ATX and other inflammatory and fibrotic mediators, which are reported to be positively modulated in COVID-19 [51–54], might help us understand the significance of this finding.

Contrary to this study, recently increased expression of ATX in immune cells and increased serum levels of ATX have been reported in the subjects with severe COVID-19 [55,56]. The discrepancies between this study and the previous study remained unknown. Considering that ATX is mainly produced from adipose tissue [37], not immune cells and serum ATX levels were higher in female subjects [29], whereas the serum ATX levels were not stratified in that study, we believe that the serum ATX decreases in the subjects with COVID-19. Moreover, this study investigated the serial changes of the serum ATX levels, compared with paired statistical methods using the serum ATX levels measured after day 21 as well as those of healthy subjects, considering that the reference range of ATX is rather wide (Figure 1).

An important limitation of this study was that we did not measure circulating LPA levels, since it requires strict plasma sampling on account of the easy release of LPA from platelets and from lysophosphatidylcholine, the substrates of ATX, present in the blood samples during preparation of the samples [57]. Moreover, a previous study reported that the plasma 18:1 LPA levels were higher in COVID-19 patients [11], although the plasma 18:1 LPA levels were higher in that study than in our recent study, in which sampling of plasma was conducted under stricter conditions [58], and the data on serial measurements and measurements on specified days after the onset of COVID-19 were not available in that study. However, considering that the serum ATX levels were positively correlated with LPA levels [29], we believe that these results of this study may shed some light into the involvement of the ATX and LPA axis in the pathogenesis of COVID-19. In this study, the healthy subjects were younger than the patients. However, we believe that the comparison with paired statistical methods using the serum ATX levels measured after day 21 could properly evaluate the modulation of serum ATX levels in COVID-19.

In summary, COVID-19 patients showed a decrease in the serum levels of ATX, an enzyme involved in the synthesis of LPA, irrespective of the disease severity. Although the pathophysiological significance remained to be fully elucidated at present. The alterations in the levels of this lysophospholipid-related protein might represent compensatory biological responses directed at suppressing immunological overreaction of the body in COVID-19, which is an important risk factor for mortality from the disease.

## Supplementary Material

Supplemental Material

## Data Availability

All the data was included in the manuscript and Supplemental Materials. The datasets generated or analysed in this study will be made available upon reasonable request. All materials are commercially available.
