# Supplementary material for: Decrease in serum levels of autotaxin in COVID-19 patients
Source: Ann Med. 2022 Nov 11;54(1):3189–200. doi: 10.1080/07853890.2022.2143554 (PMC9665086; doi:10.1080/07853890.2022.2143554)
Supplement: Supplemental Material [file IANN_A_2143554_SM0516.pdf]

Supplemental Table S1. Number of samples obtained for measurement of the serum ATX levels on specified days after the onset of COVID-19.

	**Mild**		**moderate**		**Severe**	
	male	female	male	female	male	female
**Pre**	2	3	1	1	0	0
**day 1-2**	2	1	1	1	0	1
**day 3-4**	8	3	3	2	0	1
**day 5-6**	12	5	17	6	4	1
**day 7-8**	12	12	19	6	5	3
**day 9-10**	8	9	26	8	11	5
**day 11-12**	10	7	35	10	12	4
**day 13-14**	8	7	24	11	14	5
**day 15-16**	9	2	18	4	12	3
**day 17-18**	1	1	5	6	5	2
**day 19-20**	2	0	6	3	7	2
**day 21~**	12	7	31	13	14	3

Supplemental Table S2. Number of the samples obtained on specified days after the onset of COVID-19 to compare the serum serum ATX levels measured on these days with those measured later than day 21 after disease onset.

	mild	moderate	severe
**Pre**	1	2	0
**day 1-2**	1	2	0
**day 3-4**	5	5	0
**day 5-6**	7	19	3
**day 7-8**	12	19	5
**day 9-10**	9	25	12
**day 11-12**	10	32	11
**day 13-14**	9	23	13
**day 15-16**	8	17	10
**day 17-18**	2	11	6
**day 19-20**	0	7	7

Supplemental Figure S1

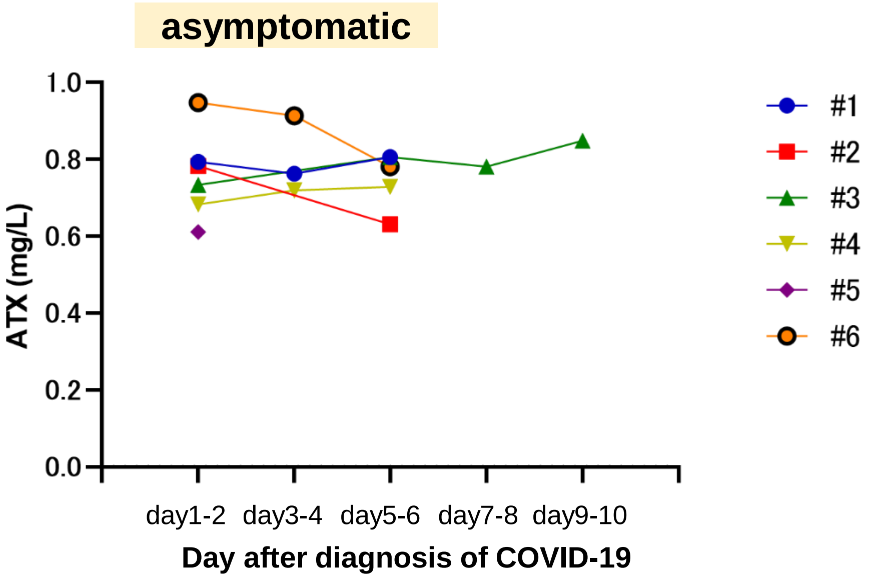

Supplemental Figure S1. Time-course of serum ATX levels in asymptomatic COVID-19 patients

Time-course of serum ATX levels in asymptomatic COVID-19 patients (n = 6). The horizontal bar represents the means of serum ATX levels measured after day 21.

Supplemental Figure S2

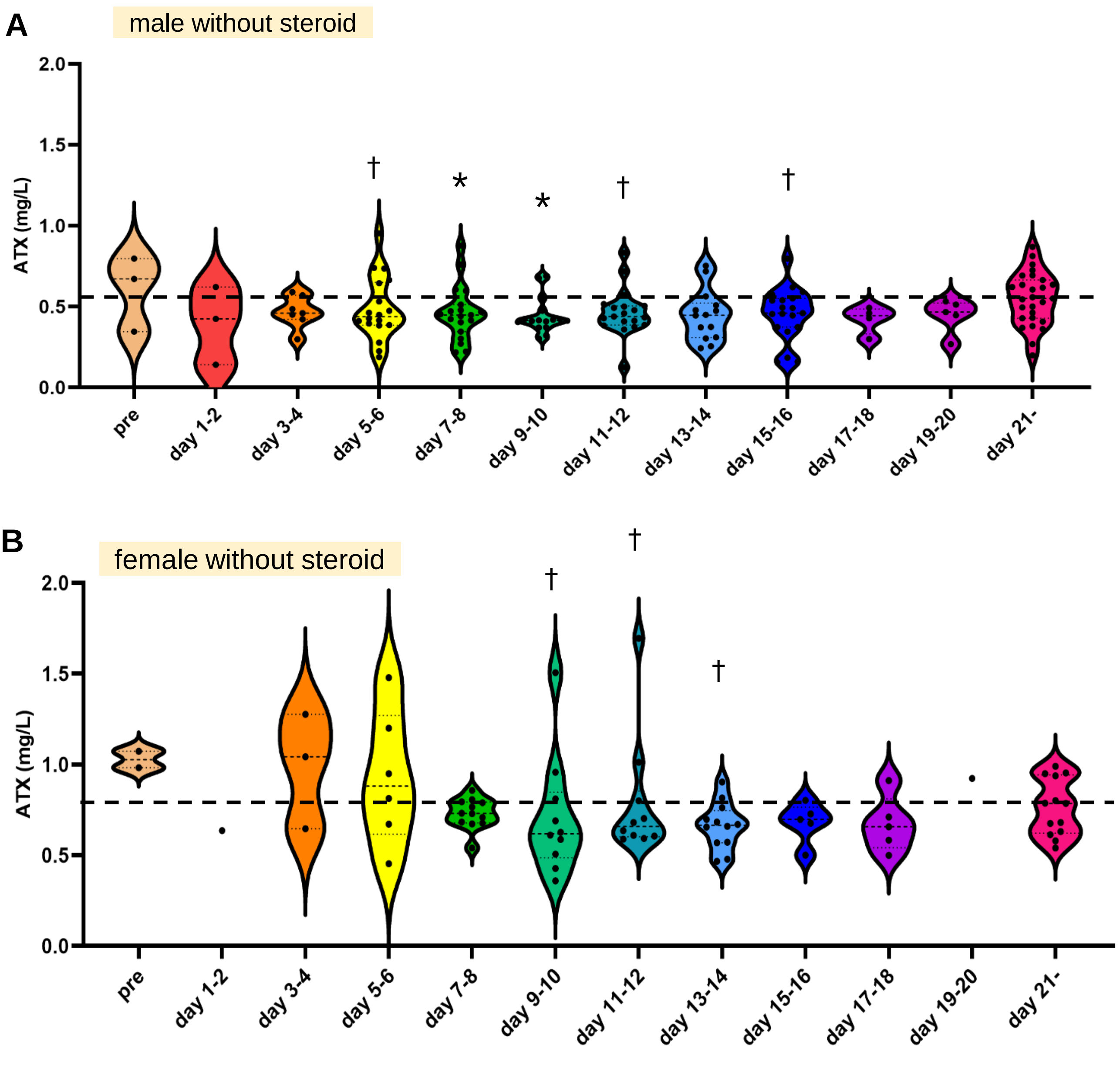

Supplemental Figure S2. Time-course of the serum ATX levels in COVID-19 not treated with steroids.

Associations between the serum ATX levels and use of steroids in COVID-19 patients are presented. (A) Male patients with COVID-19 not treated with steroids (n = 38). (B) Female patients with COVID-19 not treated with steroids (n = 24). Differences in the serum ATX levels measured on specified days after the onset of COVID-19 and those measured after day 21 in individual subjects were assessed by the Wilcoxon signed-rank sum test. * $P < 0.01$, † $P < 0.05$ vs. level measured after day 21. The horizontal bar represents the means of serum ATX levels measured after day 21.

Supplemental Figure S3

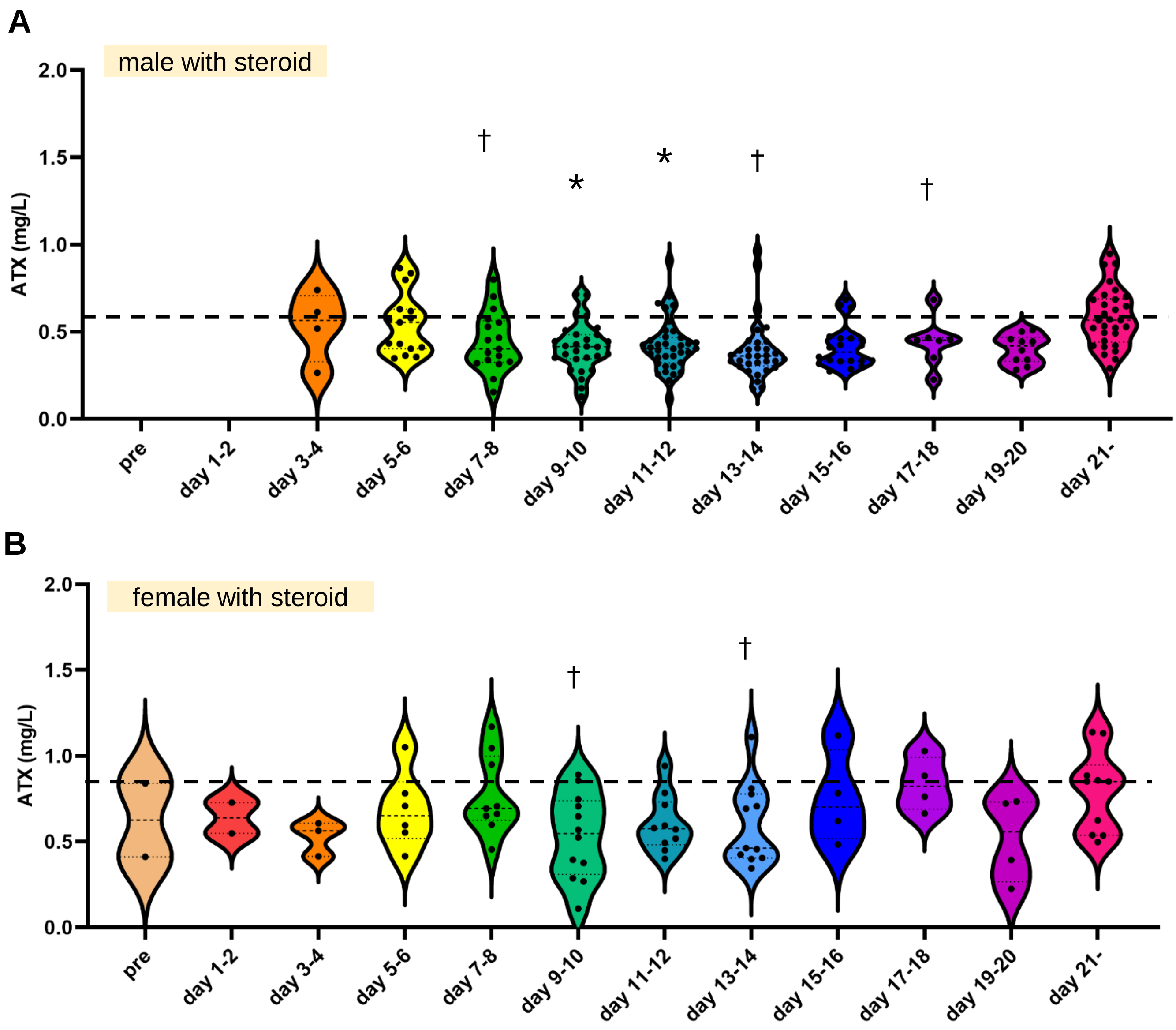

Supplemental Figure S3. Time-course of the serum ATX levels in COVID-19 patients treated with steroids.

Associations between the serum ATX levels and use of steroids in COVID-19 patients are presented. (A) Male patients with COVID-19 treated with steroids (n = 49). (B) Female patients with COVID-19 treated with steroids (n = 16). Differences in the serum ATX levels measured on specified days after the onset of COVID-19 and those measured after day 21 in individual subjects were assessed by the Wilcoxon signed-rank sum test. * $P < 0.01$, † $P < 0.05$ vs. level measured after day 21. The horizontal bar represents the means of serum ATX levels measured after day 21.

Supplemental Figure S4

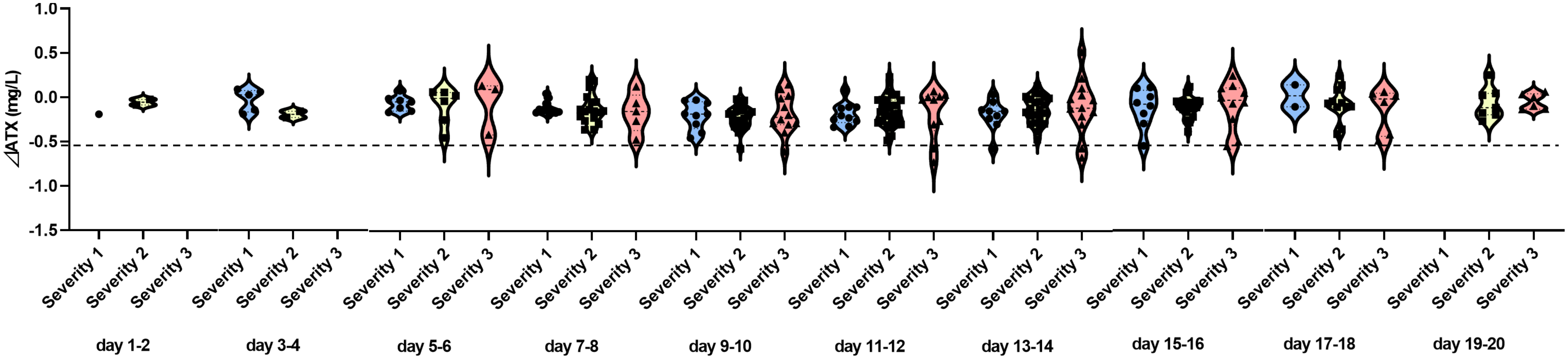

Supplemental Figure S4. Time-course of the differences between specific points and after day 21 in each maximum severity group.
The differences between specific points and after day 21 in individual subjects (⊿ATX = [ATX on specific day] – [ATX after day21]) were calculated. Differences in ⊿ATX among three maximum severity groups were assessed by an independent Kruskal-Wallis test. No significant differences were observed. The horizontal bar represents ⊿ATX = 0 mg/L.
